# Detection of myocardial oedema with the use of diffusion-weighted imaging in acute myocardial infarction

**DOI:** 10.1186/1532-429X-13-S1-P98

**Published:** 2011-02-02

**Authors:** Anna Kociemba, Magdalena Lanocha, Katarzyna Katulska, Andrzej Siniawski, Magdalena Janus, Stefan Grajek, Malgorzata Pyda

**Affiliations:** 1 1'st Clinical Hospital, University of Medical Sciences, Poznan, Poland; 2 Poznan University of Medical Science, Poznan, Poland

## Introduction

MR diffusion-weighted imaging is an important application for oedema detection in various tissues. Evaluation of the area at risk in reperfused acute myocardial infarction (AMI) is currently performed with STIR T2-weighted and LGE sequences.

## Purpose

The aim of the study was to find practical application for a new diffusion-weighted sequences in evaluation myocardial oedema and compare it with routinely used STIR-T2 techniques.

## Methods

In preliminary study myocardial oedema in 15 patients (13 male) with STEMI within 2-4 days were evaluated .The CMR examination was performed on a 1,5 T scanner (Magnetom Avanto; Siemens; Germany; Erlangen;) using a 8-channel phased-array coil. The parameters of the diffusion-weighted EPI sequence (DWI) were as follows: slice thickness 10mm, repetition time (depending on patient breath cycle) 3-4s, echo time 78ms, bandwidth 1,736 Hz/Px. The DW sequence was ECG-gated and synchronized to the respiratory cycle using PACE technique. Each slice was acquired with b = 50 s/mm2, 400s/mm2 and 800 s/mm2 with three perpendicular directions of the diffusion gradient. DW, STIR T2-weighted and LGE images were obtained in 2-chamber, 4-chamber or short-axis planes. Images were analysed quantitatively, contrast to noise ratio (CNR) of high signal (oedema) to healthy myocardium (CNR1) and high signal to blood (CNR2) were calculated. For statistical analysis a non parametric Wilcoxon test with significance level of p<0,05 was used.

## Results

The CNR were significantly higher on DWI than on STIR T2-weighted images: CNR1 (22±7 vs 12±8 p= 0,004, respectively)and CNR2 (28±10 vs 21±9, p=0,02 , respectively).

## Conclusions

Our study confirms DW EPI is feasible sequence for myocardial oedema detection with even better contrast to noise ratio than standard STIR T2 sequences.

**Figure 1:**
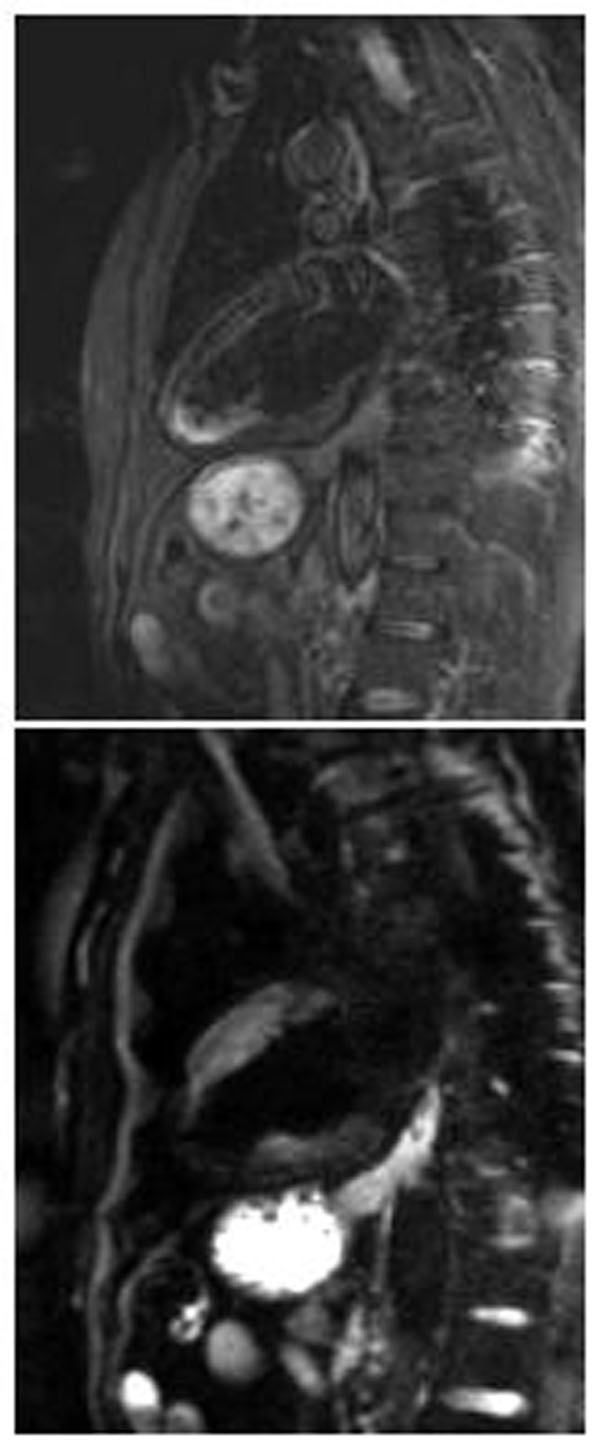
Myocardial oedema in 2 chamber view evaluated by A) STIR T2-weighted image; B) DW EPI sequence

